# Transition-metal and organocatalysis in natural product synthesis

**DOI:** 10.3762/bjoc.9.134

**Published:** 2013-06-20

**Authors:** David Yu-Kai Chen, Dawei Ma

**Affiliations:** 1 Department of Chemistry, Seoul National University, 1 Gwanak-ro, Gwanak-gu, Seoul 151-742, South Korea; 2 Shanghai Institute of Organic Chemistry, 354 Fenglin Lu, Shanghai 200032, P. R. China

**Keywords:** catalysis, natural product

The total synthesis of natural products, a field of organic chemistry that is both historical and contemporary, has undoubtedly entered a new paradigm over the past decade with the advent of revolutionary new synthetic methods and innovative synthetic concepts. Putting aside the downstream applications of natural-product synthesis in the interrogation of biological processes, elucidation of biogenetic origins, structural assignments and many others, its fundamental and indispensable value as a vehicle for the discovery of new synthetic transformations is well-testified and unparalleled by any other research discipline over the history of chemical science. These transformations, largely concerning carbon–carbon/carbon–heteroatom bond formations, asymmetric induction, and catalysis, constantly expand the repertoire of powerful tools available at the organic chemist’s disposal, and enable more challenging synthetic problems to be investigated. As such, this catalytic cycle of discovery fueled by natural-product synthesis continues to capture and captivate the imagination of both practitioners and students of organic chemistry around the world and will do so far beyond the foreseeable future. In particular, the recent discovery of novel transitional-metal complexes and their associated chemical transformations, and rediscovery of the unprecedented reactivity of previously documented transition-metal complexes with subtle changes in the reaction conditions and the reacting substrate have been extremely fruitful since the turn of the millennium, most notably in promoting reactions of unfunctionalized and unactivated chemical bonds. Furthermore, the application of organic compounds as promoters of chemical transformations has also witnessed increasing sophistication, substrate scope, and efficiency together with new modes of activation, which rival or at times surpass those exhibited by transition metals. Last but not least, the judiciary combination of transition-metal and organic mediators, together with tandem processes encompassing multiple reaction cycles in a programmed sequence, represents a new horizon with vast potentials that have yet to be fully understood and exploited.

In this Thematic Series, selected examples of metal- and organic-compound-promoted chemical processes that render the preparation of architecturally complex natural products, natural-product subdomains, or natural-product-like scaffolds, are presented. These illustrative synthetic studies are intended to showcase the most recent developments, at the same time highlight the state-of-the-art and current limitations, and in doing so set the path for the future. It is our great anticipation that this Thematic Series will instigate and inspire further investigations in this field, and challenge the existing technologies and our current mindset in target-oriented synthetic design. Ultimately, we wish that the newly acquired knowledge will translate to further advances in synthetic organic chemistry and provide more enabling tools for synthetic-chemistry-dependent research fields and beyond.

David Yu-Kai Chen and Dawei Ma

Seoul, Shanghai, June 2013

